# Supplementary material for: Pretreatment, Anaerobic Codigestion, or Both? Which Is More Suitable for the Enhancement of Methane Production from Agricultural Waste?
Source: Molecules. 2021 Jul 9;26(14):4175. doi: 10.3390/molecules26144175 (PMC8303515; doi:10.3390/molecules26144175)
Supplement: Supplementary file 1 [file molecules-26-04175-s001.zip › molecules-1279551-supplementary.pdf]

Supplementary Materials

Pretreatment, Anaerobic Co-Digestion or Both? Which is More Suitable for Enhancement of Methane Production from Agricultural Waste?

Lütfiye Dumlu[1,2], Asli S. Ciggin[1], Stefan Rucman[3] and N. Altınay Perendeci[1,*]

[1] Department of Environmental Engineering, Akdeniz University, Antalya, Turkey; asliciggin@akdeniz.edu.tr (A.S.C.)
[2] Ministry of Environment and Urbanization, General Directorate of Environmental Management, Department of Water and Soil Management, Ankara, Turkey; lutfiye.dumlu@csb.gov.tr (L.D.)
[3] Analytical Science and National Doping Test Institute (ASNDTI), Mahidol University, 272 Rama 6 Road, Bangkok 10400, Thailand; stefan.ruc@mahidol.ac.th; rucman.stefan@gmail.com (S.R.)
* Correspondence: aperendeci@akdeniz.edu.tr (N.A.P.); Tel.: +902123106334

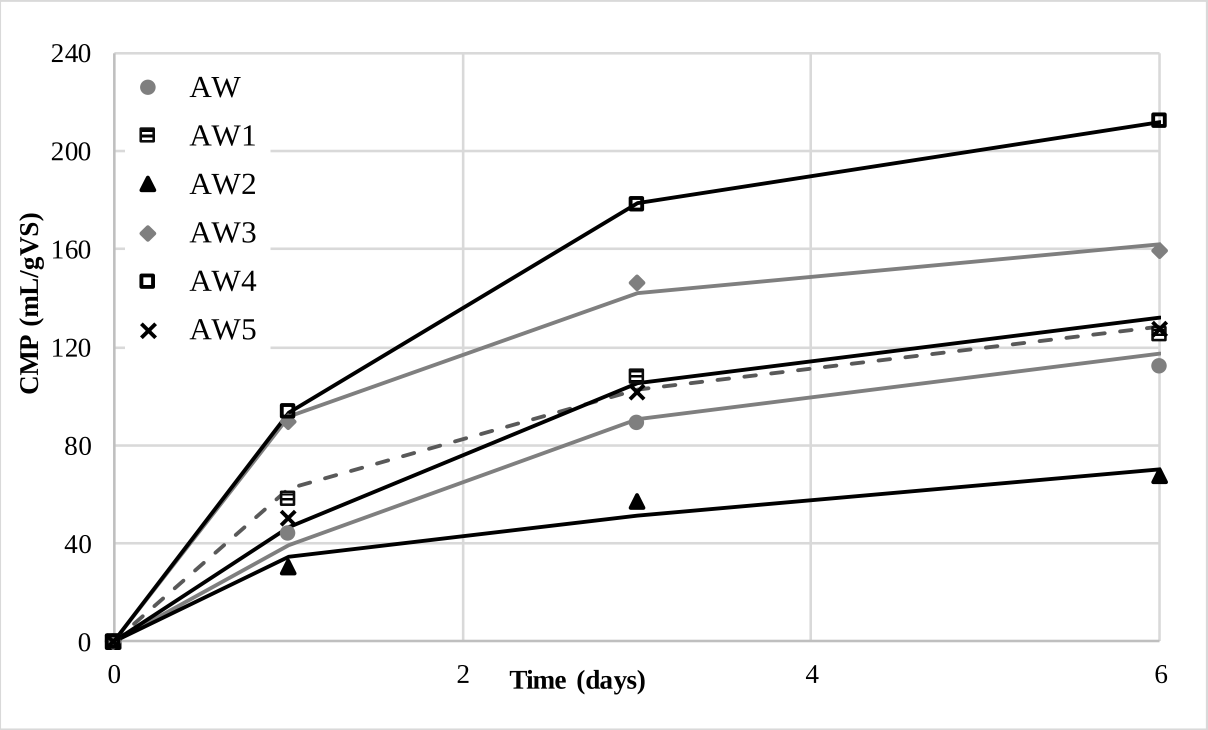

Figure S1. Model calibration of hydrolysis rates for anaerobic digestion of raw and pretreated AWs (Straight lines show experimental results; dots show model simulation results)

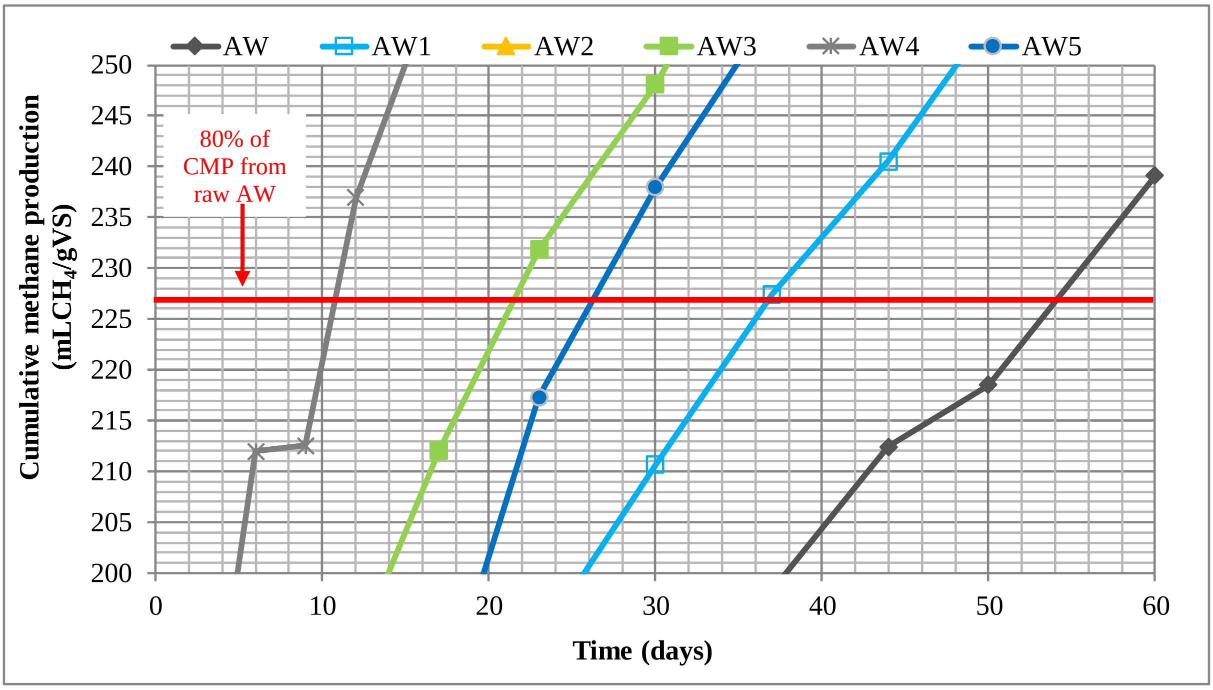

Figure S2. Determination of technical times for raw and pretreated AW

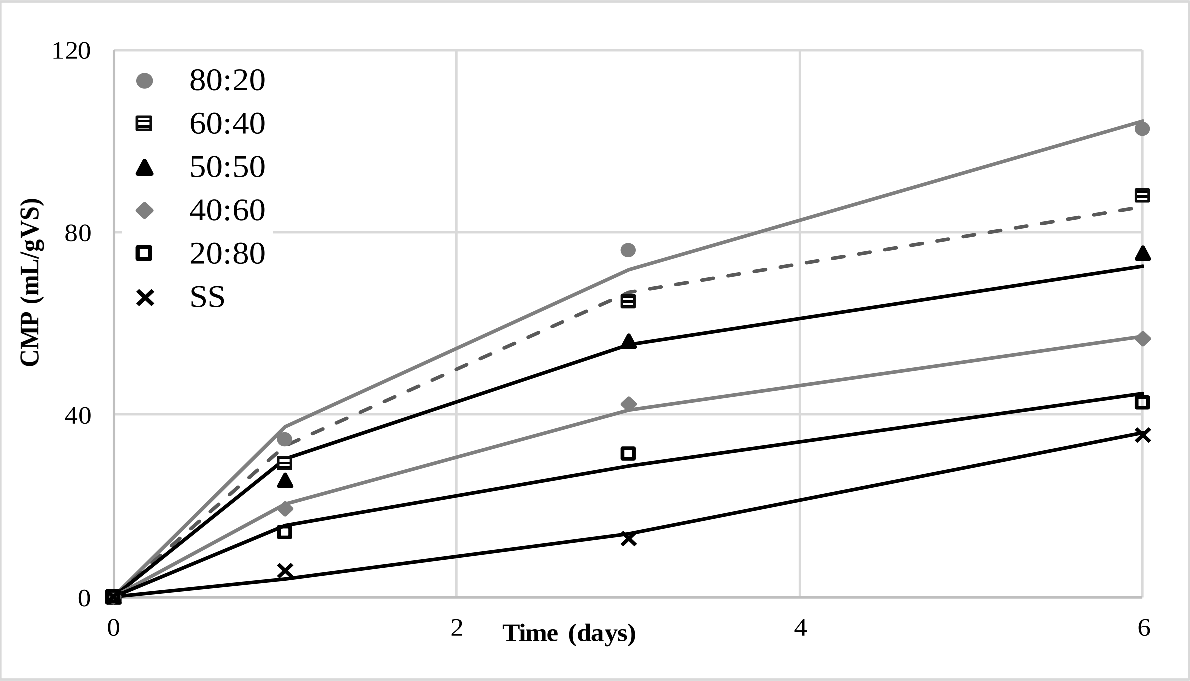

Figure S3. Model calibration of hydrolysis rates for AcoD of raw AWs with SS (Straight lines show experimental results; dots show model simulation results)

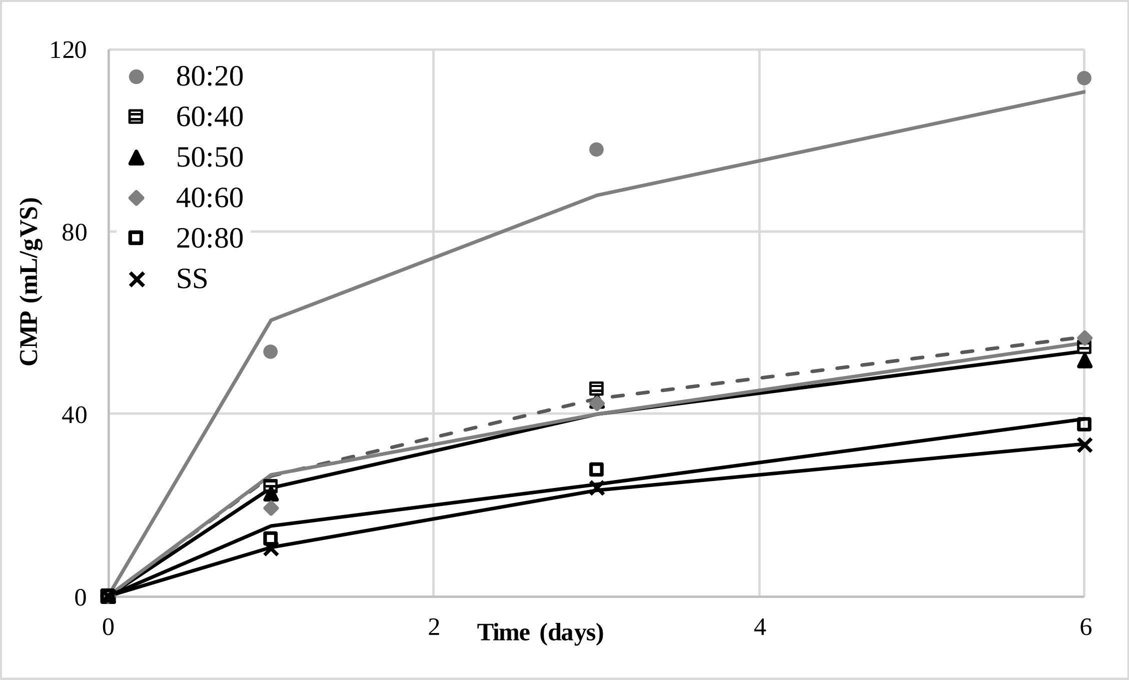

Figure S4. Model calibration of hydrolysis rates for AcoD of AW3 with SS (Straight lines show experimental results; dots show model simulation results)
